# Sex Differences in Neurological Emergencies Presenting to Multiple Urban Level 1 Trauma Centers

**DOI:** 10.1089/neur.2023.0050

**Published:** 2023-09-12

**Authors:** Linda Papa, John J. Cienki, Jason W. Wilson, Virginia Axline, Emily A. Coyle, Ryan C. Earwood, Josef G. Thundiyil, Jay G. Ladde

**Affiliations:** ^1^ Department of Emergency Medicine, Orlando Regional Medical Center, Orlando, Florida, USA.; ^2^ Department of Emergency Medicine, Jackson Memorial Hospital, Miami, Florida, USA.; ^3^ Division of Emergency Medicine, Morsani College of Medicine, Tampa, Florida, USA.; ^4^ Department of Emergency Medicine, Henry Ford Health, Detroit, Michigan, USA.; ^5^ Department of Emergency Medicine, Stony Brook University, Stony Brook, New York, USA.

**Keywords:** emergency department, neurological emergencies, migraine, seizures, sex, stroke, traumatic brain injury

## Abstract

Previous studies have suggested that there are sex differences in the treatment and outcome of neurological emergencies; however, research identifying the role these sex differences play in the management of neurological emergencies is lacking. More knowledge of the way sex factors into the pathophysiology of neurological emergencies will be helpful in improving outcomes for these patients. The aim of this cross-sectional study was to assess the prevalence and management of neurological emergencies while evaluating sex differences in the diagnosis and treatment of these emergencies. We analyzed a cohort of 530 adult patients from four level 1 trauma centers over a period of 4 weeks who had a chief complaint of a neurological emergency, including seizures, cerebrovascular events, headache disorders, traumatic brain injuries, and central nervous system infections. Among patients with neurological emergencies, a significantly lower proportion of female patients underwent neurosurgery and were admitted to the intensive care unit compared to male patients, but there were no significant differences between sexes in the time of symptom onset, type of hospital transportation, amount of neuroimaging performed, admission rates, hospital length of stay, and disposition from the emergency department. Although female patients were more likely to have a chief complaint of headache compared to traumatic injuries in male patients, this was not statistically significant. A significantly higher proportion of female patients had health insurance coverage than male patients.

## Introduction

Neurological emergencies, such as traumatic brain injury (TBI), cerebrovascular events, headache disorders, seizures, and central nervous system (CNS) infections, are a diverse group of conditions that lead to a significant number of emergency department (ED) visits each year. In recent years, a widened appreciation of sex differences in the diagnosis, treatment, and outcome of these emergencies has fueled a push for research in this area.^1,2^ Subsequently, quality evidence has accumulated supporting sex differences in neurological emergencies that warrants exploration of differences in management that may lead to better outcomes.

It has been shown that the presenting symptom profile may differ by sex in conditions like idiopathic intercranial hypertension (IIH) and bacterial meningitis.^3^ Sex hormones, such as estrogen and testosterone, have also been implicated in risk differences in a variety of conditions, including stroke^4,5^ and chronic pain.^6–9^ Disparities in treatment exist as well.^10,11^ Women tend to have longer pre-hospital delays before treatment for stroke.^12,13^ Women are also less likely to receive intravenous thrombolytic therapy (recombinant tissue plasminogen activator; rt-PA) and cerebrovascular reperfusion in acute stroke despite evidence that women may benefit more than men from the use of rt-PA.^10,14,15^

Despite few sex differences in the management of neurological emergency, outcome measures can vary widely. In IIH, men are reported to be twice as likely to develop severe visual loss as compared to women.^3^ Another example of this phenomenon is exemplified by post-stroke outcome measures. Women have worse post-stroke functional outcomes then men and are more likely to be discharged to chronic care facilities.^16^

Over the past decade, there have been a multitude of advances in the diagnosis and management of neurological emergencies, yet research into sex differences in neurological emergencies lags behind. For example, men present to the ED with TBI at a higher rate as compared to women, but research focusing on sex differences in management is sparse.^2^ Risk and cause of rehospitalization after TBI differ based on sex as well.^17^ Still, in one meta-analysis looking at sex as an indicator of prognosis in TBI, only 7% of identified research stratified data by sex.^18^ There is an awareness of gaps in the literature and a call aimed at improving patient care related to a number of neurological emergencies.^1,19^ It is clear that sex plays a role in the presentation and pathophysiology of neurological emergencies, and further research into this topic will likely improve management and outcomes of patients presenting with these problems.

The goal of this investigation is to assess the prevalence and type of neurological emergencies in patients presenting to multiple level 1 trauma centers. We assessed how these emergencies were managed and explored sex differences in diagnosis, testing, treatment, and outcome.

## Methods

### Study design and setting

This study was designed as a cross-sectional study of consecutive patients presenting to the participating ED with a neurological emergency, including with new-onset seizures, intracerebral hemorrhage (ICH), subarachnoid hemorrhage (SAH), TBI, spinal cord injury, ischemic stroke, and meningitis/encephalitis.

This multi-center study included data from four level 1 trauma centers who participate in the Florida Alliance for Research in Emergency Medicine (FLARE-EM). This research network supports the conduct of high-quality interdisciplinary, multi-center clinical research studies in EDs across Florida.

### Study population and methods

Patients were identified through ED registration logs during a 4-week period. Inclusion criteria were adults ≥18 years of age with a chief complaint of a neurological emergency. Exclusions were repeat visits during the 4-month study period by the same person and visits unrelated to neurological emergencies.

On-site abstractors at each institution underwent training before reviewing patient charts. These on-site abstractors utilized a standardized data collection form to record patient data. Data extracted from the medical records included demographic information, history of illness, and results of radiographs and neuroimaging such as computed tomography (CT) and magnetic resonance imaging (MRI) scans, electroencephalograms (EEGs), procedures and treatments received, and disposition. All sites obtained institutional review board approval before data collection. Data were collected retrospectively over 4 weeks.

Primary outcomes were sex differences in demographics, types of neurological emergencies, and presentation. Secondary outcomes included procedures and treatments such as invasive monitoring, intensive care unit (ICU) admission, and need for neurosurgery.

### Statistical analysis

Descriptive statistics were used to examine the data. Discrete data were assessed using frequencies and proportions, and continuous data were analyzed using comparative statistics such as chi-square, independent-sample *t*-tests, and the Mann-Whitney U test, together with 95% confidence intervals (CIs). Significance was set at 0.05.

## Results

There were 530 patients included in the analysis; 236 (45%) were female and 294 (55%) were male. Overall, mean age of patients with neurological emergencies was 48 years, and the racial distribution was 19% African-American/Black, 15% Hispanic, 66% Caucasian, and 1% other. Age and race were not significantly different between sex groups. A comparison of patient demographics is shown in Table 1. There were significant differences in type of medical insurance coverage between sexes, with more female patients having private insurance and Medicaid (51% vs. 37%) and more male patients without insurance and classified as self-pay (32% vs. 21%; *p* = 0.011). Although not statistically significant, female patients were more likely to have comorbidities. Characteristics of presentation, such as type of transportation to the hospital, time of symptom onset, and Glasgow Coma Scale (GCS) score, were similar between sexes. There were significantly more male patients with a history of substance abuse (22% vs. 11%; *p* = 0.001). In terms of chief complaint, there was a larger proportion of males presenting with trauma (28% vs. 22%) and a larger proportion of females presenting with headache (6% vs. 2%).

**Table 1.** Comparison of Patient Characteristics Between Sexes

	Male* N* = 294 [95% CI]	Female* N* = 236 [95% CI]	Total* N* = 530 [95% CI]	*p *value
Age, years	47 [44–49]	49 [46–52]	48 [46–50]	0.252
Race (%)				0.483
African-American/Black	52 (18)	46 (20)	98 (19)	
White	190 (65)	157 (67)	347 (66)	
Hispanic	49 (17)	29 (12)	78 (15)	
Other	3 (1)	4 (2)	7 (1)	
Had medical insurance (%)	224 (76)	196 (83)	420 (79)	0.067
Insurance type (%)				0.011
Private/HMO	64 (22)	66 (28)	130 (25)	
Auto	19 (7)	11 (5)	30 (6)	
Other	14 (5)	5 (2)	19 (4)	
Medicare	59 (20)	50 (21)	109 (21)	
Medicaid	45 (15)	54 (23)	99 (19)	
Self-pay	93 (32)	50 (21)	143 (27)	
Mean number of comorbidities (*n* = 470)	2.2 (2.0–2.4)	2.4 (2.2–2.6)	2.3 (2.1–2.5)	0.246
No. of comorbidities (*n* = 470) (%)				0.176
0	19 (7)	7 (3)	26 (6)	
1–2	156 (61)	130 (61)	286 (61)	
3–4	57 (22)	53 (25)	110 (23)	
≥5	25 (10)	23 (11)	48 (10)	
Transported by ambulance/helicopter (*n* = 527) (%)	235 (80)	178 (76)	413 (78)	0.287
Was there a delay in seeking medical attention? (*n* = 494) (%)	67 (23)	56 (24)	123 (23)	0.300
Time from symptom onset to ED presentation (hours) (*n* = 494)	12.6 [7.7–17.4]	10.8 [7.7–13.9]	11.8 [8.8–14.8]	0.572
Family or friend present (*n* = 518) (%)	138 (48)	126 (55)	264 (51)	0.111
History of substance abuse (%)	66 (22)	27 (11)	93 (18)	0.001
GCS score (%)				0.504
<8	34 (12)	19 (8)	53 (10)	
9–12	20 (7)	17 (7)	37 (7)	
13–15	180 (61)	156 (66)	336 (63)	
Not recorded	60 (20)	44 (19)	104 (20)	
Chief complaint (%)				0.067
Trauma	81 (28)	51 (22)	132 (25)	
AMS	20 (7)	20 (9)	40 (8)	
TIA/stroke	57 (19)	45 (19)	102 (19)	
Seizure	112 (38)	93 (39)	205 (39)	
Headache	6 (2)	14 (6)	20 (4)	
Syncope	7 (2%)	5 (2%)	12 (2%)	
Weakness/dizziness	5 (2%)	2 (1%)	7 (1%)	
Fever	4 (1%)	0 (0)	4 (1%)	
Pain/other	2 (1%)	6 (3%)	8 (2%)	

HMO, health maintenance organization; ED, emergency department; GCS, Glasgow Coma Scale; AMS, altered mental status; TIA, transient ischemic attack; CI, confidence interval.

In Table 2, invasive interventions, surgeries, and patient disposition across sexes are described. There were significantly more male patients who underwent neurosurgery (6% vs. 2%; *p* = 0.046) and required admission to the ICU (25% vs. 17%; *p* = 0.043) compared to female patients. Despite these differences, the proportion of female and male patients who required intubation, central line placement, and intracranial pressure monitoring was not significantly different. Admission to the hospital, hospital length of stay, and disposition from the ED to home or rehabilitation were not significantly different between sex groups.

**Table 2.** Comparison of Patient Interventions and Disposition Between Sexes

	Male* N* = 294 [95% CI]	Female* N* = 236 [95% CI]	Total* N* = 530 [95% CI]	*p *value
Admitted to the hospital (*n* = 528) (%)	181 (62)	144 (61)	325 (62)	0.928
Length of hospital stay	6.3 (5.1–7.5)	5.2 (4.3–6.2)	5.8 (5.0–6.6)	0.419
Required ICU admission (*n* = 528) (%)	73 (25)	41 (17)	114 (22)	0.043
Intubated (*n* = 526) (%)	46 (16)	27 (12)	73 (14)	0.205
Central line (*n* = 521) (%)	32 (11)	28 (12)	60 (12)	0.681
Required neurosurgery (*n* = 493) (%)	17 (6)	5 (2)	22 (5)	0.046
Required ICP monitoring (*n* = 493) (%)	8 (3)	4 (2)	12 (2)	0.560
Disposition from the ED (*n* = 528) (%)				0.362
Home/community	103 (35)	86 (37)	189 (36)	
Rehabilitation	7 (2)	3 (1)	10 (2)	
Ward	98 (33)	91 (39)	189 (36)	
Stepdown/telemetry	14 (5)	14 (6)	28 (5)	
OR	8 (3)	2 (1)	10 (2)	
ICU	61 (21)	37 (16)	98 (19)	
Deceased	2 (1)	2 (1)	4 (1)	

ICU, intensive care unit; ICP, intracranial pressure; ED, emergency department; OR, operating room; CI, confidence interval.

There were no significant differences in neuroimaging performed between sexes (Table 3). CT was performed in 80% of male patients versus 76% of female patients, MRI in 23% versus 30% respectively, cerebral angiography in 14% in both sexes, and EEG in 3% in both. Abnormalities in neuroimaging were similar between sexes. Further, presenting vital signs were comparable in both sexes (Table 4).

**Table 3.** Comparison of Neuroimaging Characteristics Between Sexes

	Male* N* = 294 (%)	Female* N* = 236 (%)	Total* N* = 530 (%)	*p *value
CT scan head performed	234 (80)	179 (76)	413 (78)	0.343
CT scan head abnormal	137 (59)	100 (56)	237 (57)	0.616
MRI brain performed	68 (23)	71 (30)	139 (26)	0.074
MRI brain abnormal	58 (85)	56 (79)	114 (82)	0.381
Cerebral angiography performed	40 (14)	33 (14)	73 (14)	0.900
Cerebral angiography abnormal	23 (59)	21 (66)	44 (62)	0.628
EEG performed	10 (3)	7 (3)	17 (3)	0.810
EEG abnormal	7 (70)	3 (43)	10 (59)	0.350
Advanced spine imaging performed	11 (4)	7 (3)	18 (3)	0.810
Advanced spine imaging abnormal	7 (58)	2 (25)	9 (45)	0.197

CT, computed tomography; MRI, magnetic resonance imaging; EEG, electroencephalography.

**Table 4.** Comparison of Presenting Vital Signs Between Sexes

	Male* N* = 294 [95% CI]	Female* N* = 236 [95% CI]	Total* N* = 530 [95% CI]	*p *value
Systolic blood pressure (*n* = 514)	142 [138–145]	141 [138–145]	141 [139–144]	0.933
Pulse rate (*n* = 518)	88 [85–90]	88 [86–91]	88 [86–90]	0.668
Respiratory rate (*n* = 506)	19 [18–19]	18 [18–19]	18 [18–19]	0.486
Oxygen saturation (*n* = 475)	98 [98–98]	98 [98–98]	98 [98–98]	0.275
Temperature (*n* = 477)	36.6 [36.5–36.8]	36.6 [36.5–36.7]	36.6 [36.5–36.7]	0.586

CI, confidence interval.

## Discussion

The substantial prevalence of patients presenting with neurological emergencies to the ED each year mandates further investigation into the diagnosis, treatment, and clinical outcomes in a way that stratifies sex. TBI, cerebrovascular events, headache disorders, seizures, and CNS infections are a few of these neurological emergencies that have demonstrated significant sex differences despite minimal available research to create guidelines and recommendations. Concurrent application of a patient-centered, sex-specific approach to management can improve all aspects of care in those presenting with neurological emergences in the ED. Although there was no statistically significant difference in chief complaint of neurological emergency between sexes in our study, there were slightly higher proportions of males presenting with trauma and TBI and more females presenting with migraines and pain.

Our data showed that 19% of both males and females had a chief complaint of a stroke, consistent with recent literature.^20^ However, globally, the lifetime risk of stroke (from age 25 years onward) is 25.1% in women and 24.7% in men, but there is substantial regional variation.^21,22^ Risk of stroke is higher in women than men among those <30 years of age, lower in women compared with men in midlife, and similar in women and men ≥80 years of age.^23^ This likely reflects changing risks in women over a lifetime, with younger women more likely to have migraines, use oral contraceptive pills, or have pregnancy-related changes.^24^

Women have been shown to present with increased stroke severity and compromised clinical presentations. In ischemic stroke, women tend to be older and have more comorbidities and multiple stroke mechanisms. In fact, there was a trend for females in our study to have more comorbidities. Women have a higher prevalence and incidence of intracranial aneurysms, and a substantially higher incidence of subarachnoid hemorrhage compared with men, whereas men have higher rates of hemorrhagic stroke.^23^ Female sex is associated with higher levels of long-term disability than men, but mortality and cerebrovascular recurrences are not significantly different.^12,25^ This is consistent with our results. Although this study did not extrapolate the specific type of complaints associated with stroke, it is documented that women present with atypical symptoms, and this reduces the timely recognition of stroke.^12,25^ Our study did examine neurological imaging between sexes and found no significant variations in abnormal CT scan of the head, MRI of the brain, or cerebral angiography.

In terms of headache as a chief complaint, 6% of females in our study presented with headaches compared to 2% of males. Females also presented with complaints of pain (3%) more often than males (1%). This is consistent with other ED data showing that women are 3 times more likely to present to the ED for a headache and ∼5 times more likely to present for a migraine as compared to that of men.^1,26^ Women are also more likely to report pain as a presenting symptom with headaches, post-concussion syndrome, and mild TBI. This has been postulated to be attributable to increased pain sensitivity in women caused by a combination of genetic, biological, and psychosocial determinants.^8,18^ Although our study did determine that females more commonly presented to the ED with the complaint of a headache, it did not reach statistical significance.

One possible explanation is that a breakdown of the type of headaches (i.e., cluster, tension, or migraine) was not available. Although previously thought of as a male-specific condition, cluster headaches have become more common in women as well, largely attributable to the changes in diagnostic criteria that have helped differentiate them from misdiagnosed migraines.^27^ The data suggest that female patients generally may be more gravely affected by a cluster headache than male patients.^28^ Despite the assumption that female changes in sex steroids, such as the withdrawal of estrogen during menstrual cycles, play a significant part in the pathogenesis of migraines, the evidence is not yet well understood and still being explored.^29^ Given the burden of headaches and migraines and its disproportionate distribution between sexes, the incidence, frequency, and intensity of headache migraine attacks must be better understood.^29^ Relevant biological as well as behavioral differences must be taken into account.

Men had significantly higher rates of substance abuse as compared to women in our study, predisposing them to conditions such as bacterial meningitis and hemorrhagic and ischemic stroke and other cardiovascular disorders.^30–33^ Those using marijuana, injectable illicit drugs, and e-cigars are at particular risk.^30–32^ Drug abusers 15–44 years of age are >6 times more likely to have a stroke than non-drug users.^32^ Alcoholism is considered an “immunocompromised” state according to Dias and colleagues, increasing a patient's susceptibility to bacterial and viral infections and causing neurological milieu.^6^ Heavy alcohol use has an increased risk of ICH in men and SAH in both sexes.^34^ Seizures are an extremely common occurrence in those with substance abuse whether attributable to direct intoxication, withdrawal, or physiological and anatomical changes in the brain.^35,36^ Alcohol has been shown to raise the seizure threshold through the flow of calcium and chloride through ion-gated glutamate N-methyl-D-aspartate and gamma-aminobutyric acid receptors with a substantial decrease after cessation, causing withdrawal seizures.^36^ Despite our findings that men had higher rates of substance abuse, our data did not show any significant increase in specific chief complaints between sexes.

Pre-hospital delays and diagnostic accuracy have been hypothesized as potential causes for variation in treatments between patients, given that there is a narrow window where intervention is possible in numerous neurological emergencies. Some studies have shown that women present later to the hospital because of their increased incidence of living alone and having an unwitnessed stroke as compared to their male counterparts.^1^ Although not statistically significant, there was a trend for more males to present by ambulance than females. Further, women have previously been identified as presenting with “stroke mimics,” complicating the picture for emergency providers to differentiate what is truly the symptoms of a cerebrovascular incident or instead another adverse neurological event.^37,38^

Our study demonstrated that males and females were both found to have similar modes of transport, delays in seeking medical attention, and time from symptom onset to ED presentation. This finding was more consistent with conclusions from Reeves and colleagues, indicating no clinically significant differences in pre-hospital delays.^24^ Our study did not analyze symptomatic presentation in detail, but vital signs and GCS did not show any statistical difference.

Although pre-hospital care showed no differences, in-hospital management was found to have significant variations. Both sexes received similar imaging studies with comparable abnormalities determined, contradictory to data showing that there was an increased use of investigational methods, such as carotid imaging and CT, for women as compared to men. Despite this, our study identified that males significantly required more frequent ICU admissions as well as eventual neurosurgical intervention compared to their female counterparts. Bias in ICU admission and surgical intervention have been highlighted in the literature, describing that women are less likely to receive advanced measures of care.^1,20^ Di Carlo and colleagues noted that a possible theory for discrepancies in management could be attributable to the misconstrued idea that women have a higher rate of morbidity or mortality in light of surgical correction.^25^

It has also been postulated that this difference could be attributable to factors not associated with sex such as patient-specific contraindications for treatment and differences in eligibility. Still, sex biases toward social roles have been thought to play a role as well, although no concrete conclusions have been established.^1,25^ Despite these findings, our study noted no significant difference in procedural interventions—including intubation, central line placement, and intracranial pressure monitoring—between males and females, but there was a trend for males to have more interventions. These findings are in line with recent literature, thus providing further support that the disparities in care for different sexes may not be significant.^24^

Studies have shown that women have worse functional outcomes after a cerebrovascular accident, including higher rates of admission to rehabilitation facilities and more difficulty performing activities of daily living. Advanced age, increased comorbidities, worse pre-stroke functional status, and less social support in women have been suggested as possible causes for this difference.^39^ However, our analysis did not reveal any significant sex-specific differences in disposition rates to either home or rehabilitation facilities. Additionally, the number of patients who died because of a neurological emergency in the ED was identical in men and women. These contradictory findings are potentially because patient demographics in our population, including patient age, race, and number of comorbidities, were not statistically different, although specific comorbidities that may predispose patients to various neurological sequala were not detailed in our study. Further, the number of patients with a friend or family member present was comparable between sexes, diverging from the sentiment that women have less social support as described in other studies.^39^

In our analysis, 24% of males had no health insurance coverage compared to 17% of women. Nationally, a higher proportion of women than men have health insurance.^40^ In a 2022 report, the uninsured rate for persons <65 years was 9.1% for women and 11.6% for men, a difference of 2.5 percentage points.^40^ Although the uninsured rate was higher in our sample, the ratio between male and female patients was similar to the national average.

There are limitations to our study that include the brief sampling period of 4 weeks. It may not have captured variations in presentation at different times of the year. We did, however, include patients from four distinct level 1 trauma centers. The sample size was relatively small given that these trauma centers see well over 100,000 patients annually.

## Conclusion

Among patients with neurological emergencies presenting to four level 1 trauma centers, a significantly lower proportion of female patients underwent neurosurgery and were admitted to the ICU compared to male patients. However, there were no significant differences between sexes in time of symptom onset, type of hospital transportation, amount of neuroimaging performed, admission rates, hospital length of stay, and disposition from the ED. Although female patients were more likely to have a chief complaint of headache compared to traumatic injuries in male patients, this was not statistically significant. A significantly higher proportion of female patients had health insurance coverage than male patients.

## Abbreviations Used

CI: confidence interval
CNS: central nervous system
CT: computed tomography
ED: emergency department
EEG: electroencephalogram
GCS: Glasgow Coma Scale
ICH: intracerebral hemorrhage
ICU: intensive care unit
IH: intercranial hypertension
MRI: magnetic resonance imaging
rt-PA: recombinant tissue plasminogen activator
SAH: subarachnoid hemorrhage
TBI: traumatic brain injury
